# Representing large-scale land acquisitions in land use change scenarios for the Lao PDR

**DOI:** 10.1007/s10113-018-1316-8

**Published:** 2018-03-29

**Authors:** Niels Debonne, Jasper van Vliet, Andreas Heinimann, Peter Verburg

**Affiliations:** 1 Department of Environmental Geography, Institute for Environmental Studies, Faculty of Science, VU University Amsterdam, De Boelelaan 1085, 1081 HV Amsterdam, The Netherlands; 2 Centre for Development and Environment (CDE) and Institute of Geography, University of Bern, Bern, Switzerland; 3 Swiss Federal Institute for Forest, Snow and Landscape Research (WSL), Birmensdorf, Switzerland

**Keywords:** Land grabbing, Land systems, Modeling, Land use change, Laos, Large-scale land acquisition

## Abstract

Agricultural large-scale land acquisition (LSLA) is a process that is currently not captured by land change models. We present a novel land change modeling approach that includes processes governing LSLAs and simulates their interactions with other land systems. LSLAs differ from other land change processes in two ways: (1) their changes affect hundreds to thousands of contiguous hectares at a time, far surpassing other land change processes, e.g., smallholder agriculture, and (2) as policy makers value LSLA as desirable or undesirable, their agency significantly affects LSLA occurrence. To represent these characteristics in a land change model, we allocate LSLAs as multi-cell patches to represent them at scale while preserving detail in the representation of other dynamics. Moreover, LSLA land systems are characterized to respond to an explicit political demand for LSLA effects, in addition to a demand for various agricultural commodities. The model is applied to simulate land change in Laos until 2030, using three contrasting scenarios: (1) a target to quadruple the area of LSLA, (2) a moratorium for new LSLA, and (3) no target for LSLA. Scenarios yield drastically different land change trajectories despite having similar demands for agricultural commodities. A high level of LSLA impedes smallholders’ engagement with rubber or cash crops, while a moratorium on LSLA results in increased smallholder involvement in cash cropping and rubber production. This model goes beyond existing land change models by capturing the heterogeneity of scales of land change processes and the competition between different land users instigated by LSLA.

**Electronic supplementary material:**

The online version of this article (10.1007/s10113-018-1316-8) contains supplementary material, which is available to authorized users.

## Introduction

Large-scale land acquisitions (LSLAs) have become a significant global land changing force since their proliferation following the 2008 food crisis (Deininger et al. 2011; Messerli et al. 2014). These transactions of relatively large tracts of land to agribusinesses, investment funds, and foreign governmental players have been welcomed as a long-overdue investment in the agricultural sector, initiating new value chains, introducing new agricultural technology, and creating employment (Arezki et al. 2011). However, others emphasize concerns over human rights, land rights, and biodiversity losses (De Schutter 2011; Cotula 2012; Davis et al. 2015). Although data on LSLA is scarce and not flawless (Oya 2013), the best-available database reports 1501 known concluded LSLAs, constituting 50 million hectares of land to be dedicated to food, energy, and industrial crops (Land Matrix Project: International Land Coalition (ILC) et al. 2018, consulted 07-02-2018; Anseeuw et al. 2013). An additional 20 million hectares constitute known intended land deals, marking the ongoing nature of the phenomenon. LSLA has globally targeted densely populated, accessible croplands, and to lesser extents also remote forestlands and moderately populated areas (Messerli et al. 2014). While neither plantation agriculture nor foreign large-scale agricultural investments are exceptional in history (Mazoyer and Roudart 2006; Baglioni and Gibbon 2013), the scale of the recent upsurge is trend-breaking (Byerlee 2014) and deserves the attention of land change scientists to study drivers, trends, and impacts of the phenomenon (Messerli et al. 2013).

The concept of land systems, human-induced transformations of ecosystems and landscapes, and the resulting changes in land cover, provides a framework for the representation of the human-environment interactions on land (Verburg et al. 2013). LSLA systems set themselves apart from more traditional trajectories such as smallholder intensification and conversion to urban land, for two reasons. Firstly, the conversion that an LSLA instigates is orders of magnitude larger than conversions related to traditional smallholder farming. In this way, LSLAs break away from the traditional approach towards studying land system dynamics, which typically frames changes as being small and incremental. However, these large-scale, abrupt conversions caused by LSLAs occur within the context of, and interact with, continuous small-scale incremental land system changes (Cramb et al. 2015). Therefore, in a LSLA context, a multi-scalar approach is necessary for the explanation of current and the projection of future land system changes. Secondly, LSLAs distinguish themselves from smallholder systems in that they are often used as a policy tool to reach development targets, such as increasing land productivity, developing land identified as idle, and extending state control over the domestic rural hinterland (Borras and Franco 2012; Lavers 2012; Cotula et al. 2014). Therefore, LSLAs are often negotiated as package deals in which the investor is expected to develop road, water, or agricultural processing infrastructure, provide employment, or create technology spillovers (Lu 2015; Schönweger and Messerli 2015). This way, LSLA can be seen as an attempt at outsourcing rural development (Peeters 2015). LSLAs produce commodities that are also produced by smallholders, making them direct competitors (Byerlee 2014). In a context of smallholder transitions to cash crops, such as maize, sugar cane, and rubber (Cramb et al. 2009; Hall 2011; Thanichanon 2015), LSLAs manifest themselves as an alternative pathway to fulfilling the same land-based demands.

The distinct nature of LSLAs described above constitutes a challenge to land change models (LCMs). In land system science, LCMs are used to study land system change processes, provide projections to inform policy makers or to perform scenario analysis (Turner et al. 2007). However, the multi-scalar approach and the specific political steering of LSLAs are not adequately represented in current LCMs. Usually, the choice of resolution in these tools reflects the scale of the processes being modeled, with pixels being the units at which conversion decisions are represented (van Delden et al. 2011). However, LSLAs instigate an interaction of small-scale, pixel level changes with large-scale changes involving multiple pixels at the same time. Furthermore, when defining the drivers of land change, it should be acknowledged that LSLAs provide more than simply the plantation products—they also potentially generate a host of effects that policy makers may either find desirable or undesirable. In recent history, countries have therefore taken on very different attitudes towards LSLA in their territory, ranging from permissive to restrictive stances, depending on the effects emphasized by policy makers (Cotula et al. 2014). Therefore, there is a need to reevaluate the way drivers are defined and land use changes are allocated in LCMs.

The objective of this paper is to represent the characteristics that distinguish LSLA dynamics in a land change modeling framework and use this model to explore different LSLA development trajectories as they interact with smallholder land use dynamics. To that effect, we build on the CLUMondo land system model (van Asselen and Verburg 2013). We augmented the CLUMondo model by adding a multi-cell allocation algorithm, which is able to convert multiple contiguous cells and thereby mimics the large-scale nature of LSLAs while preserving detail in the representation of other dynamics (e.g., smallholder agriculture or urbanization). To translate possible policies towards LSLAs (from LSLA-restrictive to LSLA-encouraging), we represent the effects of LSLA perceived by policy makers in a specific demand (driver) in our model. These perceived effects can be positive or negative depending on the scenario. To our knowledge, the resulting model is the first to explicitly simulate LSLA and its interaction with smallholder agriculture. To illustrate how LSLAs can cause different land change trajectories, we applied it for the Lao PDR, a country subject to many land acquisitions, as there is a relative abundance of data on LSLA location and types available.

## Methods

### Study area

The Lao People’s Democratic Republic (hereafter called Laos) is a lower-middle-income country in Southeast Asia of 6.8 million inhabitants (2015 situation). With an average GDP growth of 8% over the last decade, it is one of the fastest growing economies, and this growth has been driven for a third by use of water, mineral, and forest resources (World Bank 2017). Poverty eradication is high on the national agenda, but is still a challenge, especially in remote areas (Epprecht et al. 2008; World Bank 2017). Agriculture constitutes a quarter of the GDP and employs 75% of the population (2010 situation). The sector is dominated by rice-based subsistence agriculture, both as upland swidden agriculture and as permanent paddy rice fields (Schmidt-Vogt et al. 2009; FAO 2017a). However, the agricultural sector is characterized by rapid commercialization (Heinimann et al. 2013). These changes manifest themselves in both LSLAs and smallholder transitions to market-oriented crops.

LSLAs in Laos are usually granted by the government in the form of land concessions or leases. A nationwide inventory in 2010 identified 1.1 million hectares, or 5% of the territory of Laos, to be an agricultural land concession or lease (Schönweger et al. 2012), although not all of these projects are large-scale (defined in this study as larger than 100 ha). The granting of concessions and leases started in 2000 and proliferated from 2005 onwards. In a follow-up of this inventory, Hett et al. (2015) found that between 2010 and 2015, the number of concessions and leases rose by 71% in the provinces of Luang Prabang and Xiengkhouang, showing that despite moratoria in 2007 (for forestry plantations) and 2012 (for eucalyptus and rubber plantations), LSLA continued. Only 30% of projects are foreign-owned; these projects constitute 72% of the total acquired area (Schönweger et al. 2012). LSLAs intend to produce rubber, timber, and cash crops such as sugar cane, biofuel crops, and coffee.

Amidst the ongoing LSLA dynamics, changes in smallholder agriculture are drastically reshaping the Lao agricultural landscape. Smallholders are intensifying and integrating into global markets (Thanichanon 2015; Ornetsmüller et al. 2016), thereby competing in the same markets as LSLAs. The still extensive swidden landscapes are rapidly transforming to permanent agriculture. Additionally, smallholders are increasingly engaging in rubber production (Manivong and Cramb 2008; Fox and Castella 2013).

### Characterizing novel land systems in Laos in 2010

**Fig. 1:**
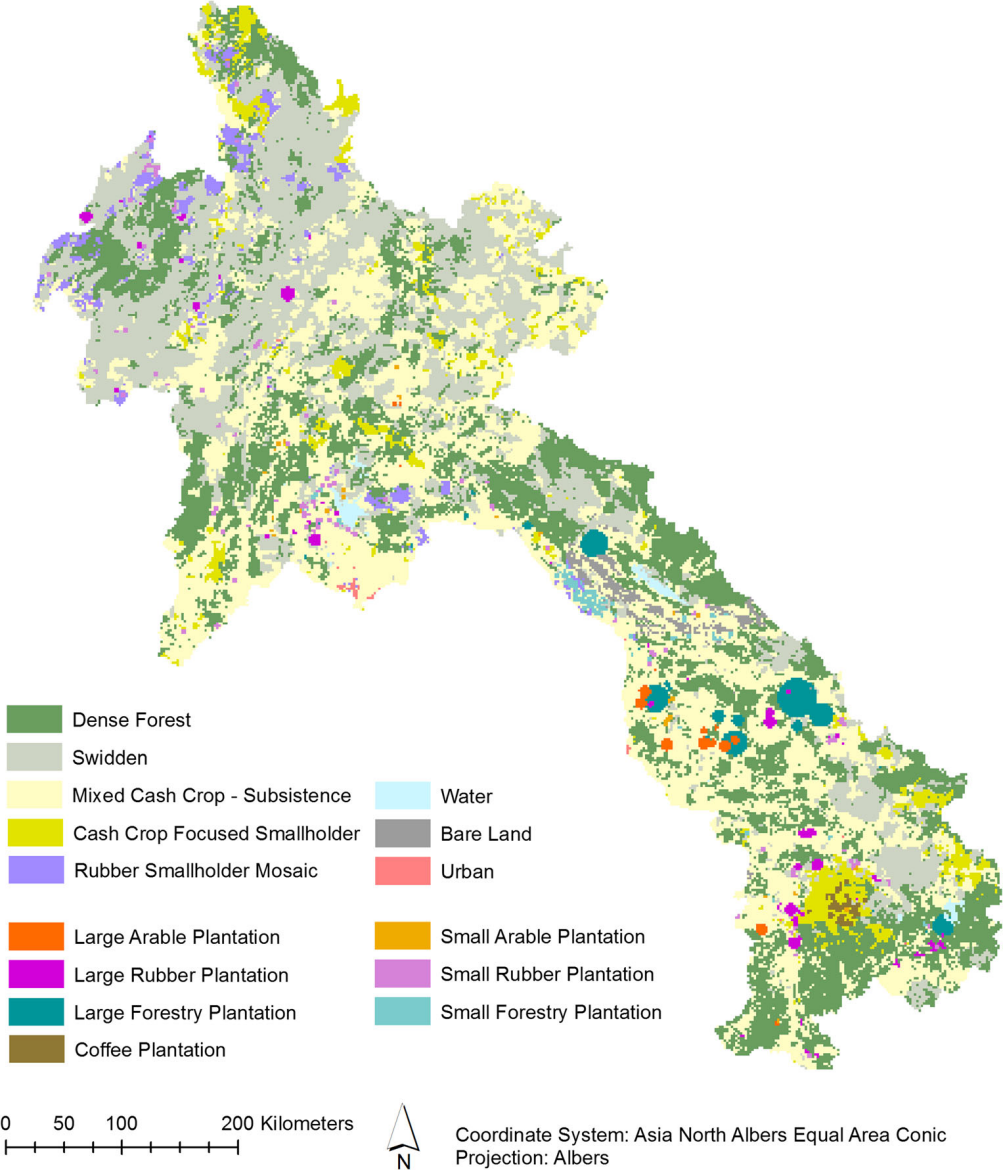
Land system map of Laos in 2010

We start our modeling exercise with a land system map representing the year 2010, based on a combination of national land cover maps, census data, and a collection of best-available data on LSLAs. All input data was first aggregated or resampled to the same spatial resolution and the same extent, to ensure consistency. We classify land systems, which denote typical combinations of land cover, land use, and land management (van Asselen and Verburg 2012), using a hierarchical decision tree (Fig. S1 in Supplementary Information (SI)) yielding 15 land systems. The characterization of swidden is based on Ornetsmüller et al. (2018). Because recent land use changes in Laos are characterized by both a rapid increase in large-scale land acquisitions and a smallholder transition to more diverse and marketable crops, we designed our classification to represent both these trajectories. An overview of all land systems is given in Table 1, and the resulting land system map is shown in Fig. 1. Given the resolution of available input data, we opted for a resolution of 2000 m. Details, data sources, and classification procedures are given in SI-2.

**Table 1:** Overview of land systems and their land system commodity production or services. Calculations and data sources in SI-2

Group	Land system	Land system commodities and services (production per 400-ha grid cell)					
		Subsistence crops	Cash crops	Rubber	Timber	Urban area	LSLA
Large-scale systems	Small arable plantation	260 ton	358 ton				1 unit
	Small rubber plantation	142 ton		81 ton			1 unit
	Small forestry plantation	237 ton			312 m^3^		1 unit
	Large arable plantation		1265 ton				1 unit
	Large rubber plantation			286 ton			1 unit
	Large forestry plantation				1100 m^3^		1 unit
	Coffee plantation	47 ton	1600 ton				1 unit
Smallholder systems	Swidden	296 ton	155 ton				
	Mixed cash crop—subsistence mosaic	426 ton	604 ton				
	Cash crop-focused smallholder	83 ton	1173 ton				
	Rubber smallholder mosaic	142 ton	345 ton	207 ton			
Urban system	Urban					400 ha	
Forest system	Dense forest						
Static land covers	Water						
	Bare land

Seven out of the 15 land systems represent LSLAs. For the remainder of this study, we define LSLA as an acquisition (transfer of use rights) of land of more than 100 ha, with the intention to use this land for agriculture or forestry. This definition includes industrial commodities such as rubber, but excludes acquisitions for mining, tourism, or special economic zones. While 100 ha is not particularly large in a global context, we use this threshold for Laos following Schönweger and Ullenberg (2009) because the average farm size in Laos is 1.6 ha (USAID 2013). Hence, by comparison, 100 ha can justifiably be considered large-scale. Spatial data of LSLA was obtained from the Land Observatory (Land Observatory Project 2017) and the Centre for Development and Environment (Schönweger et al. 2012; Hett et al. 2015). We classified LSLAs into seven systems based on their main produce—rubber, timber (e.g., teak or eucalyptus), arable crops, and coffee—and size (small and large, threshold arbitrarily set at 500 ha). As almost no coffee plantations are larger than 500 ha, all coffee plantations are included in one class. Furthermore, we distinguish four smallholder agriculture systems: (1) *swidden* (also known as shifting cultivation) is a rotational system where a short cultivation phase is alternated with a long fallow phase. The dominant crop in the cultivation phase is upland rice (Mertz et al. 2009); (2*) Mixed cash crop—subsistence mosaics* cultivate a mix of paddy rice for subsistence and other crops for market purposes; (3) In *cash crop focused smallholder* systems, farmers specialize towards marketable crops such as coffee, fruits, or sugar cane; and (4) *rubber smallholder mosaics* are systems with a large rubber component. The land system map is completed with *dense forest, urban, bare land,* and *water.*

**Fig. 2:**
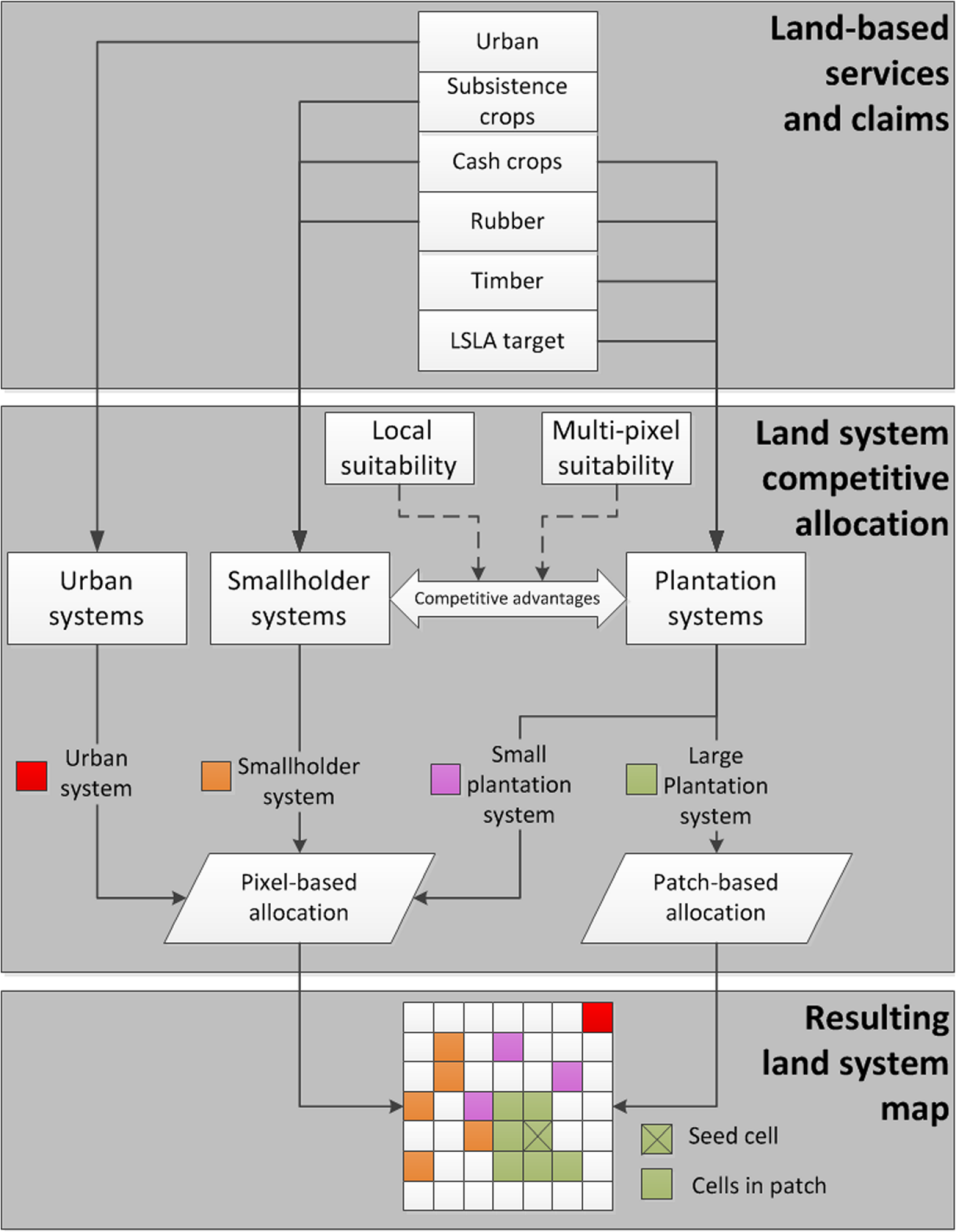
Model framework

We parameterize each land system with six commodities, services, or effects of land use it can produce in a single cell per time step (Table 1). A commodity or service can be provided by multiple land systems, and a land system can potentially provide multiple commodities, services, and effects at once, or none (e.g., water) (Fig. 2). The commodities and services are (1) *subsistence crops*, which are those crops that are predominantly produced for consumption by the producer and her family and local community; (2) *cash crops*, which are all crops except rubber that are predominantly produced for sale on regional to global commodity markets; (3) *rubber*, although also a cash crop, is represented as a separate commodity given its importance in Laos; (4) *timber*, which captures all output from forestry plantations; (5) *urban area*, representing all services the urban centers provide, including living space and infrastructure; (6) *large-scale land acquisition* itself, which is a way of quantifying the effects LSLAs are perceived to have by the host government. Whether the effects of LSLA are perceived by policy makers as positive or negative is scenario-dependent (see scenarios below). Each plantation system therefore produces one unit of “LSLA,” allowing for the definition of explicit targets on the amount of LSLAs in parameterization (e.g., a target to increase the amount of LSLAs or to cease granting of LSLAs). The empirical quantification of land system services is further described in SI-1. Note that small plantation systems also produce subsistence crops, because at the scale of a 400-ha cell, these systems are defined as a mosaic of plantations and smallholders. In contrast, large plantation systems are typified as monocultures.

The land system classification and associated commodities instigate a dichotomy between subsistence agriculture and cash crop agriculture. For smallholders, this allows the simulation of market integration, while at the same time, the competition between smallholders and LSLAs can be modeled. We empirically defined the two commodity groups based on proportions of land dedicated to cash crops, derived from the Agricultural Census (see SI-2). Commodity production figures where then calculated using typical yields reported by (FAO 2017b).

### Model description and implementation

To simulate land system changes until 2030, we applied the CLUMondo model (van Asselen and Verburg 2013). CLUMondo allocates land system changes in response to an exogenously defined demand for commodities, services, or effects in yearly time steps, using an iterative allocation procedure. In the model, alternative land systems are competing for space, based on the suitability of locations for each land system, the current land system configuration, and the competitive advantage of each system to supply the demands. The characterization of a land system includes stating the commodities, services, and effects it can provide (see previous section), the land systems it can convert into, and the system’s resistance to conversion. Yearly changes in demand for the defined land system commodities and services drive land system conversion in the model. See SI-1 and van Asselen and Verburg (2013) for an in-depth explanation of the model.

We empirically determined location suitability following the assumption that the physical and socio-economic characteristics of the current locations of land systems reflect the suitability for these systems (e.g., when more rubber is needed, rubber-producing systems will emerge in areas which have a suitable climate and/or soil for rubber tree growth and that are accessible to markets) (Van Dessel et al. 2011). The relations between these location characteristics were identified using a logistic regression analysis. We selected a set of 28 maps as candidate explanatory variables, covering climate, soil, terrain, accessibility, ethnicity, and natural hazards. Candidate explanatory variables were checked for multicollinearity, and pairs of variables that correlate too much (Pearsons *r* > 0.8) were not used in the same model. Details on variables and fitted logistic regression models can be found in SI Table S3–4.

As a consequence of the heterogeneity in scale of land change processes in a context of LSLA, a multi-scalar approach is warranted. We made two specific adjustments to the standard modeling procedures of CLUMondo: multi-cell allocation and wider-region suitability assessment (Fig. 2).

Firstly, recognizing that the large plantation systems in our application change on a multi-cellular basis, we developed a multi-cell allocation algorithm. This algorithm allocates multiple contiguous cells (patches) of a single land system, without deviating from the competition-based iteration algorithm and conversion rules. The algorithm accepts for each land system the desired patch size (stated as the maximum distance from a central cell), the minimum suitability each cell has to have to be included in a patch, and the minimum amount of cells included in each patch in order to be retained. For example, a land system can be parameterized to have patches with radius equal to 1 cell, a minimum location suitability of 0.5, and minimum number of cells included equal to 4. In that case, CLUMondo will find a seed cell at a location with high suitability for that land system and try to allocate all nine cells within the radius distance (i.e., a 3 × 3 kernel), but will be restrained by general conversion rules (e.g., water cannot be converted) and by the minimum suitability (cells with suitability lower than 0.5 for the land system will not be included). If after applying these rules, the patch has four cells or more, the patch is allocated. Otherwise, it is discarded and another location for a patch of that land system is found.

Second, when allocating large contiguous land systems, location suitability should reflect the suitability of the wider area and not simply that of a single pixel in the model. A single suitable cell surrounded by unsuitable cells is not a prime location to consider for a large-scale land system. Therefore, logistic regression models that quantify the suitability for large-scale land systems use versions of the explanatory factors that have been smoothed using a moving window focal analysis (9-cell window). Each factor has a normal layer, which quantifies a factor (e.g., flood risk) at that cell location and is used for regressions of small-scale systems, and a smoothed layer, which quantifies the average of that factor in the wider area around that cell location and is used for regressions of large-scale systems.

For our implementation of LSLAs in the model, the parameters are provided in Table 2. We set the radii and minimum amount of cells per patch to correspond to the current average size of LSLAs in Laos. The minimum suitability quantifies the extent to which the multi-cell algorithm can be selective in creating patches, and when this selectiveness is too high, no location that meets all criteria will be found. Therefore, we manually calibrated minimum suitability by adjusting it downwards until the iterative allocation procedure could find a solution.

**Table 2:** Multi-cell allocation parameters for large plantation systems

Land system	Radius (# cells)	Minimum suitability	Minimum amount of cells (400 ha) in patch
Large arable plantation	2	0.3	10
Large rubber plantation	1	0.3	6
Large forestry plantation	3	0.1	34

Because a commodity can be produced by different land systems in different quantities, a change in demand for the commodity can be resolved by seven land system change processes that summarize the possible dynamics between LSLAs and smallholders when these two producer types are in competition (visualized in Fig. S2, SI). *Smallholder intensification* occurs when one smallholder system converts into another smallholder system that produces more of the commodity (e.g., from swidden to cash crop-focused smallholder system for the cash crop commodity). Smallholder *disintensification* is the opposite (a smallholder system converts into another smallholder system that produces less of the commodity in question). *LSLA takeover* is the conversion of smallholder systems into LSLA systems, which can result in a *net gain* or *net loss* of commodity production, depending on the smallholder system that is being converted. *LSLA expansion* or *smallholder expansion* occurs when, respectively, LSLA or smallholders put dense forest systems to commodity-productive use. In our application, we restricted some trajectories that are hypothetically possible as they are deemed to be unlikely. Specifically, we restricted the conversion from LSLA to other land systems (i.e., LSLAs do not disappear), because the high capital investment and long contract times make such conversion unlikely in our time frame.

### Scenarios for land system change

We illustrate our model functionality using three contrasting scenarios of future land system change in Laos. These scenarios are characterized by (1) a high governmental encouragement of LSLA, (2) a moratorium on LSLA, and (3) no specific LSLA policy. The scenario storylines build on the notion that policy biases for or against plantation agriculture are a strong (but not the only) determinant of the occurrence of large-scale agriculture (Byerlee 2014). The scenarios are highly contrasting and serve to show a wide range of alternative trajectories, rather than a most likely future. A complete overview of all parameters and their calculations is given in the SI-4. As shown in Table 3, we assume that demands for rubber, cash crops, subsistence crops, and urban area are equal in the three scenarios. In all scenarios, it is assumed that there is an interest in LSLA in Laos, i.e., LSLA in Laos is a “seller’s market” and the amount of LSLAs in Laos can be controlled by the Government of Laos (GoL).

**Table 3:** Increase in demands of land system services until 2030 as a percentage of demand in 2010

Scenario	Timber (%)	Cash crops (%)	Rubber (%)	Subsistence crops (%)	Urban area^A^ (%)	LSLA (%)
High LSLA	160	120	200	110	182	400
Moratorium	100	120	200	110	182	100
No LSLA policy	160	120	200	110	182	n.a.

^A^Average yearly growth rate of 4.1%, based on calculations of (Ornetsmüller et al. 2016) on UN projections (United Nations 2014)

In a first scenario, “*High LSLA*", the GoL aims to include LSLA in their development strategies by granting land concessions. Policy makers thus perceive or emphasize mostly positive effects of LSLA and therefore offer attractive conditions for land investors. In the past decade, this strategy was indeed followed under the denominator of “Turning Land into Capital” and was seen as a way to increase rural accessibility to markets and infrastructure (Schönweger et al. 2012; Lestrelin et al. 2012). This scenario continues on the land capitalization track by parameterizing the model to quadruple the area of LSLAs by 2030 compared to 2010.

The second scenario, named “*Moratorium*", imposes a moratorium on new LSLAs starting from 2010. Existing LSLAs are allowed to continue operation and are not canceled. While such a moratorium has not been issued in reality in 2010, it has in 2007 (for new timber plantations) and in 2012 (for new rubber and eucalyptus plantations) (Hett et al. 2015). Scenario 2 is a stylized, extreme version of these experiences, where the moratorium encompasses all LSLAs and is assumed to be effective on the ground. Here, we assume policy makers perceive negative effects of LSLA, which they want to stop. The demand for LSLA is kept constant at the 2010 level. Timber demand is also kept constant because smallholders cannot, in our model implementation, substitute as a producer of this commodity.

The third scenario, “*No LSLA Policy*", creates a situation without restrictions or requirements for the area of new LSLAs (i.e., this specific land system effect is dropped, increasing the degrees of freedom the model has in allocating land systems). Policy makers are assumed to be indifferent and/or ineffectual towards LSLA, and do not intervene in the competitive dynamics between LSLAs and smallholders. This scenario highlights the competition between smallholders and LSLAs, and allocates land systems only based on their suitability and competitive advantages.

## Results

The three scenarios provide land system projections for Laos in 2030. After a general overview, the results from the three scenarios are presented in terms of the simulated land system changes and the processes leading to these changes.

**Fig. 3:**
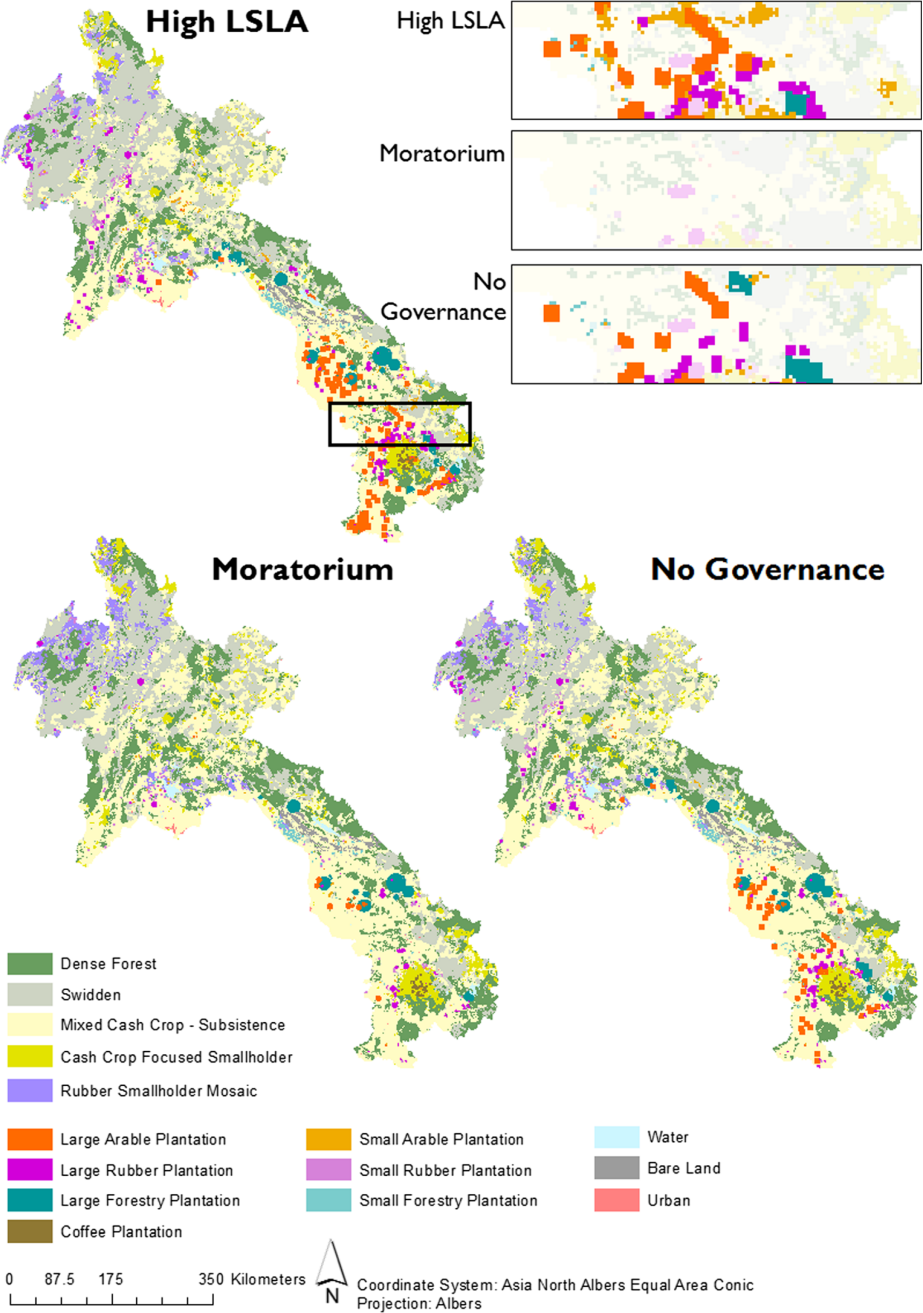
Land systems in 2030 under three scenarios. Zoomed maps visualize how scenarios differ locally and the ways in which the multi-cell allocation algorithm creates distinctively patched land systems

**Fig. 4:**
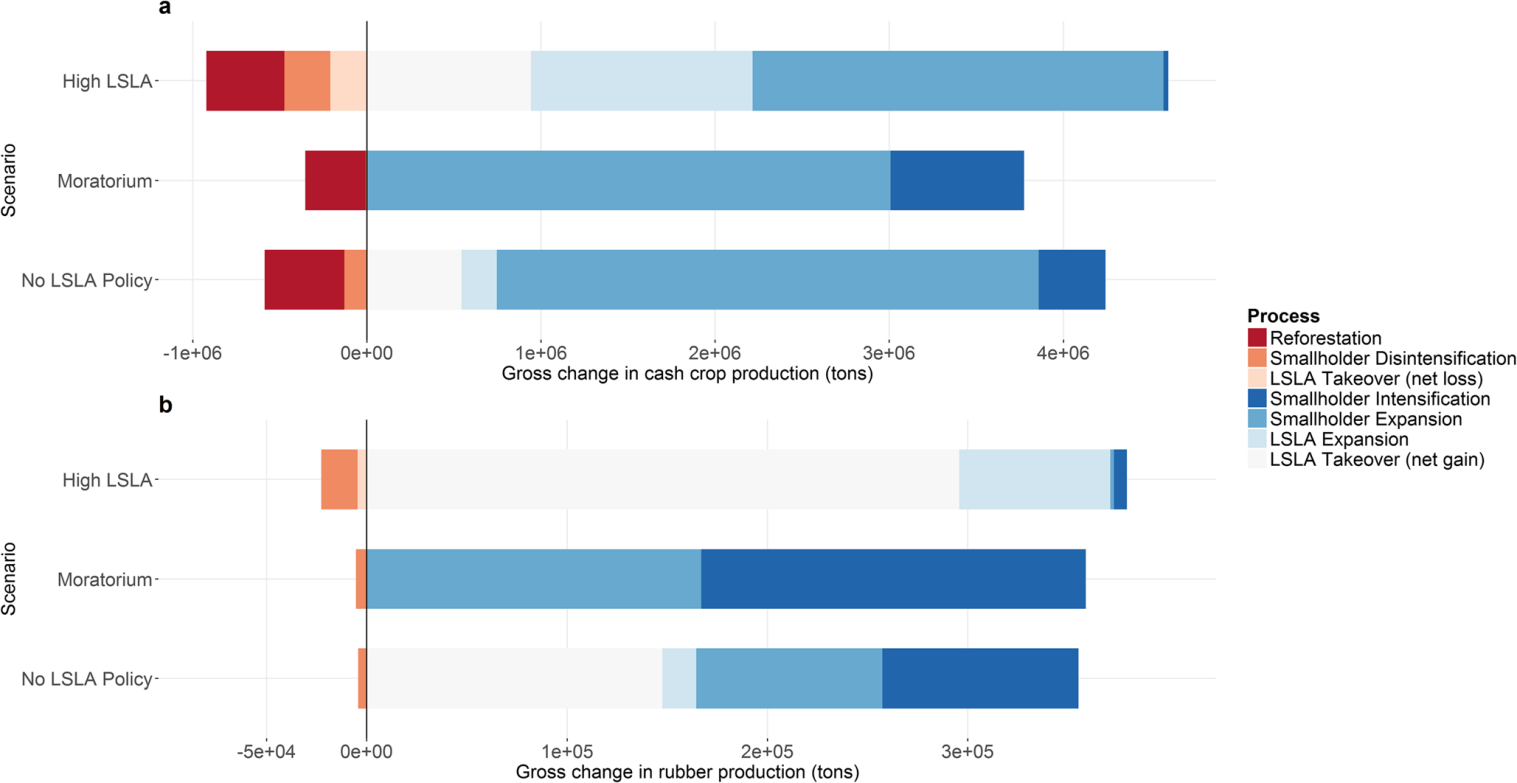
Contribution of different land system change processes to fulfilling the demand for cash crops (**a**) and rubber (**b**) in all three scenarios. Terminology for different land system change processes is given in text. The demand for both commodities is the same in all scenarios

Figure 3 shows the resulting land system maps in 2030 under the three future scenarios. The maps show three quite different land system patterns, even though the demands for most land system commodities and services are similar across all scenarios. Zoomed maps show how plantation systems are allocated, with small plantation systems allocated in the standard single-pixel mode. Large plantation systems are allocated using the multi-cell allocation algorithm, with sizes varying following Table 2. Figure 4 shows that the extent to which different land change processes contribute to the fulfillment of rubber and cash crop demands varies highly. This section describes detailed results per scenario, in terms of the simulated land system patterns and the land change processes that contribute to the fulfillment of the commodity demands.

The High LSLA scenario is parameterized to quadruple the area of LSLA by 2030. The immediate effect of this policy is proliferation of LSLAs, both by expansion into dense forest systems and takeovers of smallholder farmland. This is the only scenario where LSLA takeovers result in a reduction of cash crop and rubber production (Fig. 4). Smallholder intensification is almost non-existent in this scenario, while some disintensification takes place. Smallholder expansion is negligible for rubber, but contributes significantly to the additional cash crop production. However, this entails only conversions from dense forest to either swidden (2.01 million ha) or mixed cash crop subsistence mosaics (1.04 million ha) systems, with expansions into cash crop-focused systems being non-existent. This means that smallholders are driven to subsistence agriculture (i.e., swidden and mixed subsistence—cash crop systems), because LSLAs occupy a major part of the cash crop market as well as the land. However, these subsistence-based land systems also produce some cash crops in our model, according to the empirical characterization of these systems (Table 1). Therefore, smallholders still contribute in the provision of cash crops (Fig. 4). A surprising effect of an LSLA promotion is thus an increase in swidden extent by 18%.

The Moratorium scenario restricts LSLA proliferation, thus requiring the demands for cash crops and rubber to be met by smallholders only. Under this scenario, the Chinese border area undergoes a transformation from swidden and dense forest to rubber smallholder mosaics, and Southern Laos loses dense forest systems to mixed cash crop—subsistence mosaics. While smallholder expansion is the dominant process, this scenario also results in the most pronounced intensification by smallholders. Intensification is predominantly attained by conversions from swidden to other smallholder systems producing more cash crops and/or rubber, resulting in a net reduction of swidden extent by 11%.

In the final scenario, where no specific policy related to LSLA is in place, LSLAs supply only 18% of the increase in cash crop demand and 46% of the additional demand for rubber, compared to 48 and 98% for cash crops and rubber respectively in the High LSLA scenario. This result is significant: in the absence of policies, the land system changes are the result of the empirical characterization of land system suitability in combination with land system specific parameters. This result shows that neither smallholder nor LSLA systems are superior in terms of competitiveness in the model (i.e., the model is not significantly biased towards a specific production method). Instead, the merit of one system over the other is spatially heterogeneous. Small rubber and arable plantations are allocated significantly less in this scenario compared to the High LSLA scenario (see detail boxes in Fig. 3). This indicates that without an explicit policy demand for LSLAs, small plantation systems are only marginally competitive. Under this scenario, swidden extent decreases only by 3%.

## Discussion

### Interactions between smallholders and agricultural large-scale land acquisition

Our three scenarios show that, while the demand for rubber and cash crops can be provided by both smallholders and LSLAs, the encouragement or discouragement of LSLA results in very different spatial patterns of land system change. In our model, the distribution of the production between smallholders and LSLA depends only on the policies that govern LSLA. While LSLAs have specific economic (dis-)advantages, especially related to processing infrastructure and labor organization, policy biases for or against LSLA have historically been decisive in this distribution between LSLA and smallholder production modes in Southeast Asia (Byerlee 2014). The scenarios laid out here indicate some possible consequences of these policies on land system changes.

Results highlight that while smallholders and LSLAs are spatially segregated, they are nonetheless strongly linked. The land change processes LSLAs instigate are therefore shown to go far beyond the immediate enclosure of large tracts of land. The notion that LSLAs interact with smallholders has been identified for individual case studies (e.g., Baird and Fox 2015; Friis et al. 2016). These local studies have provided insights concerning consequences of LSLA on land systems, livelihoods, or local environments. Our study reveals larger-scale links between LSLA and smallholder agriculture through competition in common markets and land resources.

The model projects a decrease of swidden extent in the Moratorium scenario and an increase in the High LSLA scenario. Decreasing swidden extent has been a policy goal in Laos for decades (Lestrelin et al. 2013). These results highlight that swidden extent may reduce mainly through smallholder intensification processes, where smallholders increase production for commodity markets by transformation from swidden agriculture to permanent cropping, but also retain some level of production for subsistence needs. However, everything else being equal, LSLAs are projected to impede smallholder intensification and market integration, and lead to a continuation of subsistence farming, and specifically swidden farming by smallholders. Other authors have identified increased accessibility and market integration as major drivers of swidden transformations (Cramb et al. 2009; van Vliet et al. 2012). However, there are limits to converting swidden into cash crop- or rubber- producing systems, related to biophysical and cultural limitations and labor needs, making conversion to agroforestry and tree crops more likely pathways of intensification (Ducourtieux et al. 2006; Cramb et al. 2009; Vongvisouk et al. 2014). The model partly reflects these constraints using biophysical and socio-economic variables in the suitability calculations. In any case, smallholders will require organization, capital (seedlings, processing capacity), support (credit, agricultural extension programs), and infrastructure development to engage with cash crops or rubber (Ducourtieux et al. 2006; van Vliet et al. 2012). This should be seen as a prerequisite for the smallholder transformations to occur as simulated in the Moratorium scenario.

### Modeling the dynamics of agricultural large-scale land acquisition

We identified two specific characteristics of LSLAs that are relevant for their representation in land change models: heterogeneity in the scale of land change processes and the additional, policy-driven demand for the (avoidance of the) effects of LSLAs irrespective of the goods and services produced. Both are explicitly included in our presented modeling approach. The newly developed multi-cell allocation algorithm can represent the different spatial extents covered by particular land systems, which is necessary when the interaction between LSLAs and smallholder systems is addressed. The CLUMondo approach allows the inclusion of multiple demands for goods and services that drive land system changes. However, the presented application is the first in which demands for specific types of land systems are included, in addition to the still existing demand for agricultural commodities.

The multi-cell allocation algorithm gives adequate flexibility to simulate LSLAs with varying sizes (see for example the difference between large arable, forestry, and rubber plantations in zoomed maps, Fig. 3). The minimum suitability threshold can furthermore be used to simulate how much attention is given to land suitability in including individual pixels inside LSLAs, where a low threshold indicates an “anything goes” attitude, while a high threshold reflects that some attention is given to the quality of individual pixels. Unless more is known about underlying processes, the choice of these settings is arbitrary.

Simulation results are shaped by the amount of change and the location of these changes, and uncertainties or inaccuracies may appear in both (van Vliet et al. 2016). A crucial modeling step is linking current land system locations with underlying factors that determine the location choice. In the case of LSLAs, relatively little is known about location choice (Messerli et al. 2014) and our empirical analysis is based on a relatively low number of plantations (396 projects split up in seven land systems) covering a low number of cells per system. The pixels involved are, due to the patch character of LSLAs, highly autocorrelated and regression models may suffer from overfitting. Nevertheless, the approach is well suited to embed empirical evidence into the parameterization of the model. Similarly, because the exact delineation of LSLA in Laos is not known precisely, the values for the production of commodities might be over- or underestimated as well. Ongoing efforts to delineate granted, surveyed, allocated, and ultimately developed area (Hett et al. 2015) can serve to fine-tune such analysis.

Results indicate that, in all scenarios, the majority of the increase in production of rubber and cash crops may be attained by cropland expansion (to mixed extents by smallholders and LSLAs), entailing the loss of dense forest. While this signals that the commercial pressure on land may endanger current forests, the extent of this deforestation cannot be directly read from the land system change maps. A land system should be interpreted as a mosaic of various land covers, of which tree cover is one. Therefore, systems other than dense forest also contain tree cover, and net tree cover loss is contingent on the mosaic compositions. For example, LSLAs are often underused and therefore LSLA systems likely contain significant shares of forest cover (figures of productive use in this study: SI-2).

In our model, we assume that the governance of LSLA, or lack thereof, does not affect the national-level demand for commodities. However, while cash crops and rubber can be produced by smallholders as well as LSLA, their production does not necessarily respond to the same market demand. Countries and companies acquiring land are often specifically looking to control large tracts of land or speculate on future use. This interest in the control over land itself, rather than the specific land-based commodities, is referred to as “control grabbing” (Borras and Franco 2012; Hall et al. 2015) and may limit the assumed interchangeability between smallholder and LSLA production.

Differences and interactions between LSLA and smallholder agriculture in our model are to the extent possible based on existing literature. Some hypothetical differences and interactions have not been included. Firstly, there is an ongoing debate on whether the advantages of a larger-scale trump the disadvantages. Large operations are arguably better at organizing supply to a processing plant or pioneering a crop in a new area, while smallholders enjoy significantly lower costs of labor management, and often acquire higher yields due to higher-precision management for different crops (Byerlee 2014; Cramb et al. 2016). Empirical studies on this debate indicate that throughout Southeast Asian history, there has been a transition from large-scale to small-scale agriculture, making the recent surge in LSLA an aberration (Bissonnette and De Koninck 2017). Our model does not explicitly include any (dis-)economies of scale in the production distribution of crops (see Deininger and Byerlee (2012) and Hall (2011) for an in-depth discussion). Second, we have not included potential synergies between LSLA and smallholders (e.g., contract farming schemes). In such schemes, plantations may offer capital, technique, and marketing, while smallholders provide labor and land (Shi 2006; Cramb et al. 2016). However, how and to what extent such synergies result in land change processes is unclear and could be addressed in future research.

### Implications for model-based land change assessments

Since 2007, LSLA has globally become a significant land system change trajectory (Nolte et al. 2016). The interactions between LSLA and smallholders have been studied in local case studies (e.g., Friis et al. 2016; Hall et al. 2017). However, interactions at a larger scale have received far less attention (but see Baird and Fox 2015). Smallholders around the globe are stepping up as producers for the world markets of rubber, biofuel crops, and other cash crops, responding to the same global demands as LSLAs (Fox and Castella 2013; Bissonnette and De Koninck 2015; Cramb et al. 2015). The current study highlights the different potential roles of LSLA and smallholders in land system change trajectories under different scenarios. Rather than aiming at predictions of the future, these scenarios form a boundary object for discussing the option space for governments in dealing with high pressures on their land-based commodity markets and the different land system futures that may emerge from such choices, without forming normative judgments. Whether rubber and cash crop demand are met by smallholders, LSLAs, or a combination of both makes a strong difference in the emergent landscapes and the future of rural livelihoods.

Given the high impact LSLAs have on livelihoods, commodity markets, biodiversity, and forest cover, globally, it is paramount to include them in model-based land change assessments. Building sophisticated scenarios of LSLA dynamics will continue to be challenging given their regime shift-nature (Müller et al. 2014). Additionally, these systems respond to global commodity prices, which can be hard to predict. At the same time, LSLA-agnostic projections may lead to naive projections of future land change dynamics that ignore the changes in agency governing land change.

A few challenges remain. Firstly, it is widely reported that many allotted LSLAs are not actually planted or abundant for reasons of low commodity prices, local resistance, or speculative intentions of the land investor (Liao et al. 2016). Therefore, land system changes simulated here will in many cases be merely a legal change, while actual land cover change could be limited or restricted to deforestation. More detailed, local scale assessments could provide further insights in these dynamics. Furthermore communities that have been expropriated or otherwise affected by LSLAs may give rise to indirect land use changes. These lower-scale impacts on livelihoods and labor are thus a key to further understanding the impacts of LSLAs in general (Li 2011; Oberlack et al. 2016), and on land system changes specifically.

## Electronic supplementary material

ESM 1 (PDF 443 kb)
