# Supplementary material for: Representing large-scale land acquisitions in land use change scenarios for the Lao PDR
Source: Reg Environ Change. 2018 Mar 29;18(6):1857–69. doi: 10.1007/s10113-018-1316-8 (PMC6434982; doi:10.1007/s10113-018-1316-8)
Supplement: Supplementary file 1 — (PDF 443 kb) [file 10113_2018_1316_MOESM1_ESM.pdf]

Supplementary Material of: "Representing large-scale land acquisitions in land use change scenarios for the Lao PDR"
Regional Environment Change
Debonne, N.*, Vliet, J. van, Heinimann, A., Verburg, P.H.

*Environmental Geography Group, Institute for Environmental Studies & Department of Earth Sciences, Faculty of Sciences, VU University Amsterdam, The Netherlands, n.debonne@vu.nl, tel: +31 (0)20 59 87294

1: Functionality of the CLUMondo model

CLUMondo is a land system change model that can simulate land system changes in response to demands for land-based goods, services, and effects, biophysical and socio-economic variables, and land system characteristics. A complete overview of the functionality of this model is provided in Vliet and Verburg (2018) and Asselen and Verburg (2013). Here, we provide a short overview with emphasis on the functionality used in this paper.

Changing demands for land system goods, services, and effects are resolved by CLUMondo by allocating or removing land systems that can provide these demands, as defined in the land system definition and classification (see this supplement, part 2). It does this in an iterative procedure where the land system with the highest transition potential at that time and location is allocated. The transition potential of land systems producing demands that are not met are increased and vice versa, after which the procedure is repeated in a next iteration. Transition potential adjustments continue until all demands are met within a 10% error margin, and the average deviation of all demands is lower than 5%.

Transition potential is determined by the sum of three land system specific factors: (1) location suitability, (2) conversion resistance, and (3) competitive advantage. We describe these factors consecutively below.

Location suitability is quantified using logistic regressions, performed in this study using R software. 28 explanatory biophysical and socioeconomic factors have been collected from various data sources (see this supplement, part 3). These factors are given in two versions: a normal version and a smoothed version used to assess suitability for large-scale systems. Location suitability quantifies how suitable a certain location is for a certain land system. For each land system, a set of explanatory factors that relate significantly to the current location of that land system is selected. For example, in this study, we found that the current location of small rubber plantations are located on location accessible to cities and towns, with higher precipitation, and a low hazard of river flooding (see this supplement, table S4). By using logistic regression models, suitability can be quantified between 0 and 1 using these significant factors.

Conversion resistance quantifies the resistance a land system has to being converted to another land system and is related to, among others, capital investment. For example, urban land systems are typically highly resistant to change, while forest systems are more easily converted. Conversion resistance is a value between 0 and 1 and is quantified based on expert judgement (see this supplement, table S7).

Competitive advantage is initially zero for all land systems, but changes upwards or downwards during iterations. When the demands that a certain land system provides are not met, the parameter is adjusted up, and when there is overproduction of these demands, it is adjusted down. This continues until all demands are met within the defined margin of error (10% in this application and 5% on average over all demands). Note that CLUMondo does not rely on a predefined hierarchy to handle competition between different demands (one demand is not more important than another).

CLUMondo is furter constrained by the conversion matrix. Not all changes are allowed (e.g. water is not allowed to change to anythin else), and some changes take a minimum number of years to be allowed (e.g. reforestation takes a number of years). These settings are given in this supplement, table S5.

Conversion order ranks for every land system how competitive it is to deliver a specific demand. When a demand is not met, the competitive advantage of the system with the highest rank for that demand is increased more than the competitive advantge of lower-ranking systems. This ensures logical trajectories of change. Land systems can also have a negative rank (-1) for a specific demand, which means that they are not considered to resolve a deviation in demand, even though they may produce that demand (for example, in this application, small plantation systems also produce subsistence crops because they are, at the scale of the 400ha cell, in a mosaic with smallholder farmers. However, an increase in demand for subsistence crops should not result in the allocation of more small plantations). The conversion order is given in this supplement, table S6.

2: Land system classification and land system commodity and service calculations

Table S1: Classification input data

**Parameter**	**Source**	**Spatial resolution**	**Time**
Land Cover – Bare land, Water, Urban, Tree Cover	Government of laos (2010); Government of Laos (2002)	Polygons	2010 / 2002
Large-scale land acquisitions	Land Observatory Project 2017; Hett (2015); Land Observatory Project (2017); Schönweger, Heinimann, and Epprecht (2012)	Points / polygons	Updated until 2015
Upland Rice Ratio	Agricultural census 2010/11 (Ministry of Agriculture and Forestry 2014)	Village	2011
Rubber area fractions	Agricultural census 2010/11 (Ministry of Agriculture and Forestry	Village	2011

	2014)		
Cash crops area fractions	Agricultural census 2010/11 (Ministry of Agriculture and Forestry 2014)	Village	2011

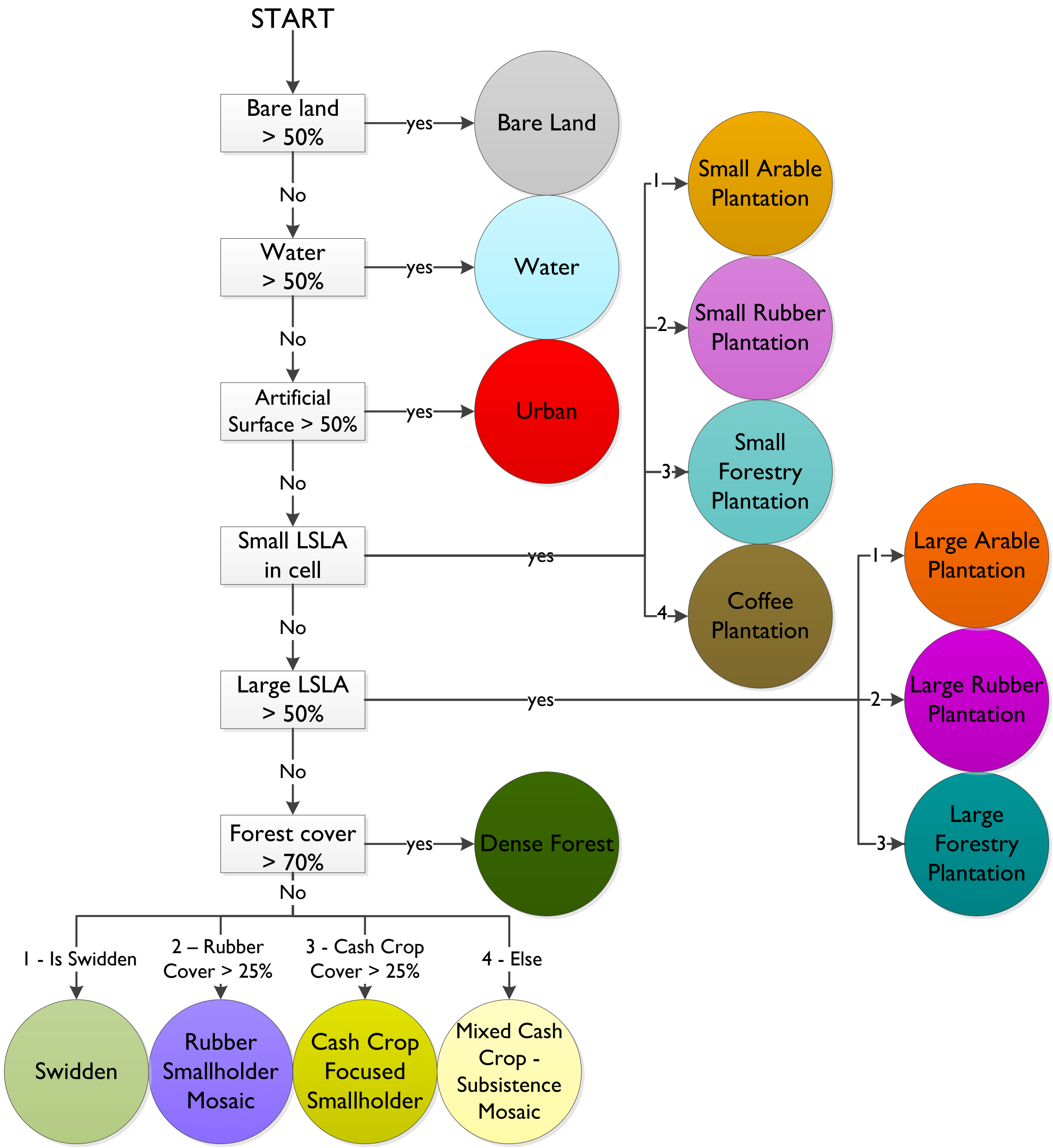

Fig S1 Land system classification decision tree. Decision tree used to map land systems. Data

sources and threshold justification in appendix 1. Numbers indicate priorities (e.g. a cell is first checked for swidden, then for rubber smallholder mosaic, etc.)

Large-scale land acquisitions data pre-processing

LSLA data was collected from the Land Observatory (www.landobservatory.org) and the Centre for Development and the Environment (CDE, Switzerland). In order to avoid errors of commission (inclusion of LSLAs that do not exist) we cross-checked the two input datasets, and consulted Google Earth satellite imagery in case of doubt. Reported LSLAs that lacked spatial accuracy (e.g. only province reported) were dropped. 396 LSLAs were thus identified. For 306 data entries, only point data was available. In this case, we created buffers of the reported area around the centroids to approach the shape. For 90 data entries, the boundary of the LSLA was available as polygon data, as part of an on-going survey (Hett 2015).

Smallholder agriculture classifications

In the land system classification decision tree, a cash crop focused smallholder system is defined as a cell where the fraction of the area that is covered by cash crops is larger than 25%. For this purpose, the sum of area fractions as reported in the Lao Agricultural Census 2010 for the following crops is calculated.

- Coffee
- Tea
- Cabbage
- Cucumber
- Avocado
- Banana
- Cashew
- Durian
- Lemon
- Longan
- Mango
- Pineapple
- Plum
- Tamarind
- Sweet corn
- Cassava
- Sugar Cane
- 'Other Fruits'

Similarly, smallholder areas where rubber covers over 25% of the area are classified as rubber – permanent smallholder mosaic. For swidden, we reclassified the land system map by Ornetsmüller, Verburg, and Heinimann (2016). This map represents a number of swidden systems with differing intensities and forest cover, which we reclassified to a single swidden system.

Land system service calculations

For all 15 land systems, the provision of each of five land system services (timber, rubber, cash crops, subsistence crops and urban area) was quantified. These services remain constant in all scenarios, whereas the LSLA service is scenario-dependent. Exception to this is the timber service in the Moratorium scenario which is kept constant, because smallholders cannot substitute as a supplier in our application.

In a first step, area breakdowns of land covers per land system were empirically established. In the cases of urban, water, dense forest and bare land, it was assumed that these land systems are covered 100% by their respective land covers. The same assumption was used for all large plantation systems. For small plantation systems (including coffee plantations), overlay analysis using the actual polygons of the plantation and the plantation land system cells was performed to determine the average area percentage of LSLAs within LSLA land system cells. Similarly, the area dedicated to cash crops by smallholders was quantified by overlaying the agricultural census (GoL, 2011) with the land

system raster. The same operation was used to calculate average tree cover of land systems, using the national land cover map (GoL 2010). Subsistence crops were calculated as the remainder area for each land system.

For LSLA in Laos, it is known that the granted area (the polygons used in this study) is often much larger than the allocated area, which is again larger than the developed area. An inventory for two Lao provinces indicates that current LSLAs use only 49% (Luang Prabang) and 12% (Xiengkhouang) of their granted area (Hett 2015). Expansion beyond the granted area also occurs but is much more rare. As there are no nationwide statistics, we quantified productive use for LSLAs by overlaying a forest map (GoL, 2010) with arable plantation polygons. This way we established that, for small arable plantations, on average 18% of the granted area is covered with trees and therefore not used productively. For large arable plantations, 45% is similarly not used productively. As the same method cannot be used for rubber, coffee or timber plantations, as the land cover map records these land uses as tree cover, we used the same number for all small and large plantations respectively, where coffee plantations are considered small plantations.

Next, typical yields were used calculate the average services output per land system. These yields are assumed to be constant across all cells belonging to the same system.

Table S2: Yields used to calculate land system commodity production

Service	Quantity	Provided by	Source and procedure
**Cash crops**	4.31 $ton.ha^{-1}yr^{-1}$ (smallholders)<br>5.75 $ton.ha^{-1}yr^{-1}$ (plantations)	All smallholder systems,<br>Arable plantations,<br>Coffee plantations	Area-weighted average for the main cash crops: permanent crops (excl. rubber), maize, sugar cane, cassava and paddy rice. Paddy rice weight adapted to count only 20% as cash crop. Yield figures obtained from (FAO 2017). Lower yield for smallholder reflect presumed lower access to inputs and technology.
**Rubber**	1100 $kg.ha^{-1}.yr^{-1}$ (smallholders)<br>1300 $kg.ha^{-1}.yr^{-1}$ (plantations)	Rubber plantations,<br>Rubber permanent smallholder mosaic	Typical values from Manivong & Cramb (2008). Higher yields for LSLAs reflect presumed better technology.
**Timber**	5 $m^3.ha^{-1}.yr^{-1}$	Forestry plantations	Typical mean annual increment yield value for Laos for eucalyptus from (FAO 2016)
**Subsistence crops**	2.6 $ton.ha^{-1}.yr^{-1}$	All smallholder systems,<br>All small plantations,<br>Coffee plantations	Weighted average of 1.7 $ton.ha^{-1}yr^{-1}$ (typical for low-input upland rice, Saito et al., 2006) and 3.59 $ton.ha^{-1}yr^{-1}$ (paddy rice, FAO 2017)
**Urban**	400 ha per cell	Urban	Cell is fully used for urban

3: Logistic regression

Table S3: Candidate explanatory factors used in the logistic regressions

Num	Variable	Abb-reviation	Original resolution	Source	Procedure
1	Elevation (m)	Elev.	90 m	SRTM[#]	Aggregated to 2000m
2	Slope (Degrees)	Slope	90 m	*Own processing*	Calculated from Elevation<br>Aggregated to 2000m
3	Terrain Ruggedness Index (m/m)	TRI	90 m	*Own processing*	Calculated using GDAL from Elevation<br>Aggregated to 2000m
4	Annual Precipitation (mm)	P_{annual}	30 arcsec	worldclim.org*	Aggregated to 2000m
5	Mean temperature	T_{annual}	30 arcsec	worldclim.org*	Aggregated to 2000m
6	Precipitation in the driest month	$P_{DriestMonth}$	30 arcsec	worldclim.org*	Aggregated to 2000m
7	Minimum temperature in the coldest month	$T_{ColdestMonth}$	30 arcsec	worldclim.org*	Aggregated to 2000m
8	Maximum temperature in the warmest month	$T_{WarmestMonth}$	30 arcsec	worldclim.org*	Aggregated to 2000m
9	Available water storage capacity (mm/m)	AWC	Region-dependent	Harmonized World Soil Database[†]	Resampled to 2000m
10	Soil drainage (5 classes)	/	Region-dependent	Harmonized World Soil Database[†]	Resampled to 2000m
11	Topsoil gravel content (%)	Topsoil gravel	Region-dependent	Harmonized World Harmonized World Soil Database[†]	Resampled to 2000m
12	Topsoil sand content (%)	Topsoil sand	Region-dependent	Harmonized World Soil Database[†]	Resampled to 2000m
13	Topsoil silt content (%)	Topsoil silt	Region-dependent	Harmonized World Soil Database[†]	Resampled to 2000m
14	Topsoil clay content (%)	Topsoil clay	Region-dependent	Harmonized World Soil Database[†]	Resampled to 2000m
15	Subsoil gravel content (%)	Subsoil gravel	Region-dependent	Harmonized World Soil Database[†]	Resampled to 2000m
16	Subsoil sand content (%)	Subsoil sand	Region-dependent	Harmonized World Soil Database[†]	Resampled to 2000m
17	Subsoil silt content (%)	Subsoil silt	Region-dependent	Harmonized World Soil Database[†]	Resampled to 2000m
18	Subsoil clay content (%)	Subsoil clay	Region-dependent	Harmonized World Soil Database[†]	Resampled to 2000m
19	Ethno-linguistic family (4 families)	/	Village	Population census 2005 (GoL 2005)	Resampled to 2000m
20	General accessibility (travel time to village centers, hours)	Gen. Accessibility	50m	Centre for Development and Environment, Bern	Aggregated to 2000m
21	Domestic market	Dom.	50m	Centre for	Aggregated to 2000m

	accessibility (travel time to district capitals, hours)	Accessibility		Development and Environment, Bern	
22	International market accessibility (travel time to district border crossings, airports, province capital, hours)	Int. Accessibility	50m	Centre for Development and Environment, Bern	Aggregated to 2000m
23	Population density	PopDens	Village	Population census 2005 (GoL 2005)	Resampled to 2000m
24	Distance to the Chinese border (km)	Dist. to China	2000m	*Own processing*	
25	Distance to the Lao country border	Dist. to border	2000m	*Own processing*	
26	River flood hazard (cm of flood with 100 year return interval)	/	1000m	Global Risk Data Platform (UNEP, 2016)	Aggregated to 2000m
27	High landslide hazard	/	0.5 arcmin	Global Risk Data Platform (UNEP, 2016)	
28	US bomb dropping density	US Bomb	Point data	US Department of Defense records‡	Heath map at 2000m resolution

USGS (2004)

*Fick and Hijmans (2017)

†Nachtergaele et al. (2009)

‡Available at https://mangomap.com/blog/delving-into-us-bombing-data-1965-1975/

Table S4: Resulting logistic regression models

Land System	Contributing factors	AUC
**Urban**	Gen. Access (+)	0.993
**Small Arable Plantation**	Dom. Access (+); P_{annual} (+); River flood hazard (-)	0.839
**Small Rubber Plantation**	Dom. Access (+); T_{annual} (+); $P_{DriestMonth}$ (+); Dist. to China (-)	0.839
**Small Forestry Plantation**	Dom. Access (+); Topsoil gravel (+); Dist. to border (-)	0.829
**Large Arable Plantation**	River flood hazard* (-); Slope* (-)	0.963
**Large Rubber Plantation**	Int. Access* (+); AWC* (+); Lao-Tai* (-); $T_{ColdestMonth}$* (+)	0.811
**Large Forestry Plantation**	Int. Access* (+); T_{Annual}* (+); P_{Annual}* (+); Poorly drained soil* (-)	0.749
**Dense Forest**	Gen. Access (-); PopDens (-); Lao-Tai (+)	0.704
**Swidden**	Gen. Access (+); P_{Annual} (-); Lao-Tai (-); Slope (+); Topsoil gravel (+)	0.752
**Rubber Smallholder Mosaic**	Dom. Access (+); P_{Annual} (+); Dist. to China (-)	0.900
**Cash Crop -Focused Smallholder**	Gen. Access (+); T_{Annual} (-); P_{Annual} (+); Topsoil clay (+); AWC (+)	0.730
**Mixed Cash Crop - Subsistence Mosaic**	Gen. Access (+); Lao-Tai (+); Population Density (+)	0.686

*Average of 3x3 cell neighborhood

4: Model parameters

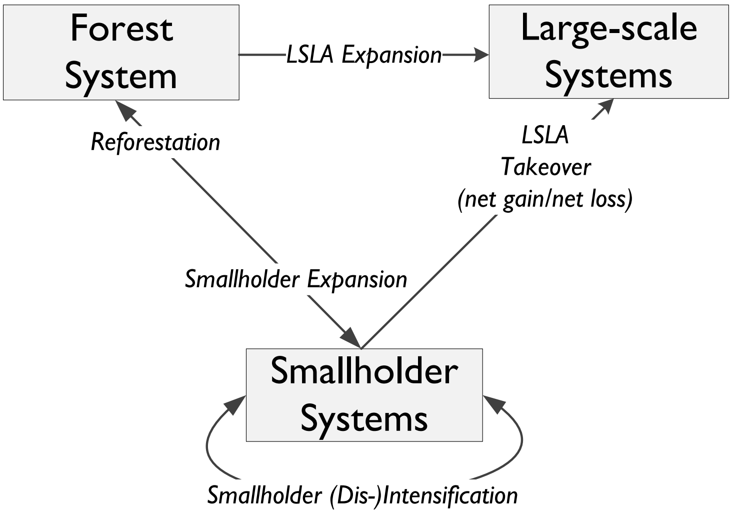

Fig. S2 Processes through which changes in demand for cash crops or rubber are resolved in the CLUMondo application. Arrows indicate processes that are allowed in the application, while other processes being restricted in our model. See main text for a description of each of these processes.

Table S5: Allowed land systems changes matrix. This table sets which land system changes are allowed (indicated by a Boolean 0 or1). The 102 in the reforestation column indicates that this change is allowed after at least 2 years. More information can be found in CLUMondo documentations at www.environmentalgeography.nl.

TO

FROM	Water	Urban land	Small Arable Plantation	Small Rubber Plantation	Small Forestry Plantation	Large Arable Plantation	Large Rubber Plantation	Large Forestry Plantation	Coffee Plantation	Dense Forest	swidden	Rubber Permanent Smallholder Mosaic	Cash Crop-Focussed Smallholder	Mixed Cash Crop - Subsistence Mosaic	Bare Land
Water	1	0	0	0	0	0	0	0	0	0	0	0	0	0	0
Urban land	0	1	0	0	0	0	0	0	0	0	0	0	0	0	0
Small Arable Plantation	0	0	1	0	0	0	0	0	0	0	0	0	0	0	0
Small Rubber Plantation	0	0	0	1	0	0	0	0	0	0	0	0	0	0	0
Small Forestry Plantation	0	0	0	0	1	0	0	0	0	0	0	0	0	0	0
Large Arable Plantation	0	0	0	0	0	1	0	0	0	0	0	0	0	0	0
Large Rubber Plantation	0	0	0	0	0	0	1	0	0	0	0	0	0	0	0
Large Forestry Plantation	0	0	0	0	0	0	0	1	0	0	0	0	0	0	0
Coffee Plantation	0	0	0	0	0	0	0	0	1	0	0	0	0	0	0
Dense Forest	0	1	1	1	1	1	1	1	0	1	1	1	1	1	0
Swidden	0	1	1	1	1	1	1	1	0	102	1	1	1	1	0
Rubber Permanent Smallholder Mosaic	0	1	1	1	0	1	1	0	0	102	0	1	1	1	0
Cash Crop-Focussed Smallholder	0	1	1	1	0	1	1	0	0	102	0	1	1	1	0
Mixed Cash Crop - Subsistence Mosaic	0	1	1	1	0	1	1	0	0	102	0	1	1	1	0
Bare Land	0	0	0	0	0	0	0	0	0	0	0	0	0	0	1

Table S6: Conversion order matrix. This table indicates the relative competitive advantages of land systems to provide land system services. Increasing numbers indicate higher competitive advantage. A 01 indicates that the land system is not considered when increasing or decreasing total supply to meet demands. The LSLA service is not used in the Moratorium scenario.

**Land system**	**Timber**	**Arable Cash Crops**	**Rubber**	**Subsistence Crops**	**Urban Land**	**LSLA**
Water	-1	-1	-1	-1	-1	-1
Urban land	-1	-1	-1	-1	1	-1
Small Arable Plantation	-1	1	-1	-1	-1	1
Small Rubber Plantation	-1	-1	1	-1	-1	1
Small Forestry Plantation	1	-1	-1	-1	-1	1
Large Arable Plantation	-1	2	-1	-1	-1	1
Large Rubber Plantation	-1	-1	2	-1	-1	1
Large Forestry Plantation	2	-1	-1	-1	-1	1
Coffee Plantation	-1	-1	-1	-1	-1	-1
Dense Forest	0	0	0	0	0	0
Swidden	0	0	0	2	0	0
Rubber Smallholder Mosaic	0	0	2	0	0	0
Cash Crop Focussed Smallholder	0	2	0	0	0	0
Mixed Cash Crop - Subsistence Mosaic	0	1	0	1	0	0
Bare Land	-1	-1	-1	-1	-1	-1

Table S7: Conversion resistance. This table indicates the resistance a land system has to changing into a different land system.

Land System	Conversion resistance
Water	1
Urban land	1
Small Arable Plantation	0.8
Small Rubber Plantation	0.8
Small Forestry Plantation	0.8
Large Arable Plantation	0.9
Large Rubber Plantation	0.9
Large Forestry Plantation	0.9
Coffee Plantation	1
Dense Forest	0.4
Swidden	0.3
Rubber Smallholder Mosaic	0.7
Cash crop-Focussed Smallholder	0.7
Mixed Cash Crop - Subsistence Mosaic	0.5
Bare Land	1

References

Asselen S van, Verburg PH (2013) Land cover change or land-use intensification: Simulating land system change with a global-scale land change model. Glob Chang Biol 19:3648–3667. doi: 10.1111/gcb.12331

FAO (2017) FAOSTAT. In: FAOSTAT Food Agric. data. http://www.fao.org/faostat. Accessed 6 Oct 2016

FAO (2016) Annual volume increment in Eucalyptus camaldulensis plantations in different parts of the world. www.fao.org/docrep/004/ac121e/ac121e04.htm. Accessed 6 Oct 2016

Fick, S.E. and R.J. Hijmans, 2017. Worldclim 2: New 1-km spatial resolution climate surfaces for global land areas. International Journal of Cli Fick, S.E. and R.J. Hijmans, 2017. Worldclim 2: New 1-km spatial resolution climate surfaces for global land areas. International Journal of Climatology.

GoL (2005) Population Census 2005. Vientiane

Government of laos (2010) Land Cover Assessment.

Government of Laos (2002) land Cover Assessment.

Hett C (2015) Land Deals In Laos : First Insights From A New Nationwide Initiative To Assess The Quality Of Investments In Land.

Land Observatory Project (2017) Land Observatory. www.landobservatory.org. Accessed 6 Mar 2016

Manivong V, Cramb RA (2008) Economics of smallholder rubber expansion in Northern Laos. Agrofor Syst 74:113–125. doi: 10.1007/s10457-008-9136-3

Ministry of Agriculture and Forestry (2014) Lao Census of Agriculture 2010/11 - Analysis of Selected Themes. Vientiane

Nachtergaele F, van Velthuizen H, Verelst L, et al (2009) Harmonized World Soil Database. Rome

Ornetsmüller C, Verburg PH, Heinimann A (2016) Land system change in the Lao PDR: scenarios of transitions from shifting cultivation to permanent cropping and tree plantations. Appl Geogr 75:1–22. doi: 10.1016/j.apgeog.2016.07.010

Saito K, Linquist B, Atlin GN, et al (2006) Response of traditional and improved upland rice cultivars to N and P fertilizer in northern Laos. F Crop Res 96:216–223. doi: 10.1016/j.fcr.2005.07.003

Schönweger O, Heinimann A, Epprecht M (2012) Concessions and Leases in the Lao PDR: Taking stock of land investments, 1st edn. Geographica Bernensia, Vientiane

UNEP (2016) Global Risk Data Platform. http://preview.grid.unep.ch. Accessed 3 March 2016

USGS (2004), Shuttle Radar Topography Mission, Global Land Cover Facility, University of Maryland, College Park, Maryland

Vliet J van, Verburg PH (2018) A Short Presentation of CLUMondo. In: Camacho Olmedo MT, Paegelow M, Mas JF, Escobar F (eds) Geomatic Approaches for Modeling Land Change Scenarios, 1st edn. Springer, pp 485–492
